# Probable Encephaloid Disease of the Left Kidney

**Published:** 1872-11-01

**Authors:** J. A. Hamilton

**Affiliations:** Odin, Ills.


### PROBABLE ENCEPHALOID DISEASE OF THE LEFT KIDNEY.

REPORTED BY J. A. HAMILTON, M. D. ODIN, ILLS.

I was requested on Monday, 3d inst., by Dr. J. J. Fyke of Odin, to accompany him to Mr. Harris, about two miles south of their farm, to assist him in the *post mortem* examination of a child, aged about eight years, who had died the day previous (as was diagnosed by Dr. Fyke), with a tumor of the abdomen; but Dr. Patten of Walnut Hill (a regular knight of the steam tub, pepper and lobelia), who had been called in consultation with Dr. Fyke during the illness of the child, pronounced the disease a simple abcess of the bowels; recommended that it should be punctured, its contents evacuated, and that the patient would recover. Dr. Fyke differed with Dr. Patten in opinion, and hence the *post mortem*.

We made an incision, commencing at the *regio*-hypogastrica, carrying it along the middle line of the abdomen to the sternum, one other incision beginning just above the navel, and extending it around the left side to the spine, thus laying bare the anterior portion of the tumor. There was nothing remarkable or particularly interesting about the tumor except its immense size; it weighed eight (8) pounds.

For the satisfaction of Dr. Patten, and those who might incline to his diagnosis, Dr. Fyke punctured the tumor in several places, but it discharged nothing but a little serum, and it was adherent to all parts with which it came in contact as the transverse colon, sigmoid flexure, peritonium, and walls of the abdomen. There was no particular base of attachment by which it grew, and was supported by the general system. Its external surface was smooth and regular, and seemed perfectly organized, but upon making a section of it, we found the organized substance about one half inch thick—the remainder, consisting of a broken down, disorganized mass, resembling a powder partially wetted. There was no kidney, or anything resembling one on that side. The other organs were all sound and in healthy condition. Death resulted from pressure upon the diaphragm, obstructing thereby the action of the lungs.

